# Simplified UNICORN for Transcatheter Aortic Valve Replacement

**DOI:** 10.1016/j.jaccas.2026.108252

**Published:** 2026-05-13

**Authors:** Raviteja R. Guddeti, Janelle Muuse, Santiago Garcia, Richard Bae, Nadia El-Hangouche, Puvi Seshiah

**Affiliations:** The Carl and Edyth Lindner Research Center, The Christ Hospital, Cincinnati, Ohio, USA

**Keywords:** coronary obstruction, leaflet modification, transcatheter aortic valve replacement

## Abstract

**Objective:**

The UNICORN (undermining iatrogenic coronary obstruction with radiofrequency needle) technique represents an emerging leaflet modification strategy for patients undergoing transcatheter aortic valve replacement (TAVR) at risk for coronary obstruction. We describe a simplified UNICORN procedure in patients undergoing valve-in-valve and native TAVR.

**Key Steps:**

This approach employs radiofrequency energy to traverse the base of the prosthetic aortic leaflet, followed by serial balloon inflations to create a large fenestration and subsequent transcatheter heart valve deployment, removing the risk of leaflet obstruction.

**Potential Pitfalls:**

Technical challenges include severely calcified leaflets that impede balloon advancement, the potential for leaflet fragment embolization with resultant coronary artery occlusion, and acute aortic insufficiency after balloon laceration.

**Take-Home Message:**

Compared with BASILICA (bioprosthetic aortic scallop intentional laceration to prevent iatrogenic coronary artery obstruction), UNICORN offers potential advantages, including enhanced leaflet tissue displacement and procedure simplification, minimizing the need for wire exchanges and snare retrieval maneuvers.

## Graphical Abstract

**Figure:**
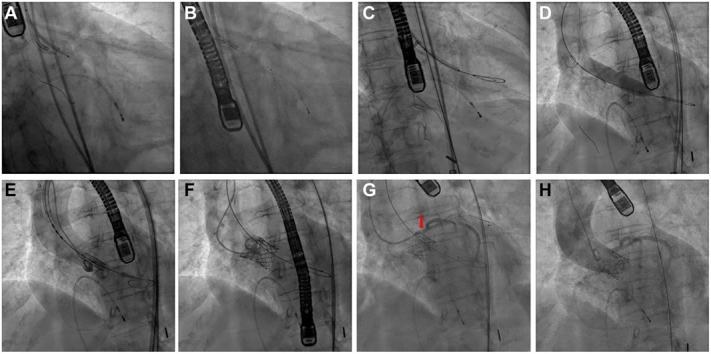

Iatrogenic coronary obstruction is an uncommon but life-threatening complication after transcatheter aortic valve replacement (TAVR), associated with significant morbidity and mortality. The incidence of coronary obstruction during TAVR is about 0.7% to 3.5%, with a higher incidence seen in valve-in-valve (ViV) TAVR, as demonstrated by data from the Global Valve-in-Valve Registry.^1^ The risk is higher in stented bioprosthesis with externally mounted leaflets and stentless bioprosthesis compared with stented bioprosthesis with internally mounted leaflets.^2^ Several strategies have been proposed to prevent coronary obstruction, including chimney/snorkel stenting and BASILICA (bioprosthetic aortic scallop intentional laceration to prevent iatrogenic coronary artery obstruction). The adoption of BASILICA is limited by procedural complexity, and inadequate leaflet splitting is another concern of BASILICA. The ShortCut device (Pi-Cardia) is a dedicated leaflet splitting device that was evaluated in ViV TAVR, and evidence regarding its use in native TAVR is limited.^3^

**Take-Home Messages**

• Compared with BASILICA, the simplified UNICORN offers potential advantages, including enhanced leaflet tissue displacement and procedure simplification, minimizing the need for wire exchanges and snare retrieval maneuvers.
• Key determinants of success include meticulous preprocedural planning, identification of calcium-free traversal targets, and coordinated multimodality imaging guidance and risk assessment.

The UNICORN (undermining iatrogenic coronary obstruction with radiofrequency needle) technique is an alternative coronary protection strategy first proposed by Chan et al^4^ in a patient undergoing ViV TAVR. In the initial description of UNICORN, a 0.035-inch VersaCross J-tip radiofrequency (RF) wire (Baylis/Boston Scientific) was used to traverse the valve leaflet, followed by exchanging the wire with a 300-cm long coronary wire for balloon dilatations; this wire was eventually exchanged for a long Safari wire (Boston Scientific) for TAVR.

The initial UNICORN technique requires multiple wire exchanges, and acute aortic regurgitation from leaflet laceration may result in hemodynamic instability. In the following 2 cases, we describe a simplified UNICORN technique in patients undergoing ViV TAVR and native TAVR.

## Case Summary 1

A 69-year-old man with a history of surgical aortic valve replacement with a 25-mm Mosaic valve in 2015, chronic heart failure with preserved ejection fraction, and end-stage renal disease on hemodialysis presented to the hospital with hypotension after dialysis. Echocardiogram revealed bioprosthetic aortic valve dysfunction with severe aortic stenosis as the mode of failure. Given his high surgical risk, ViV TAVR was recommended by the heart team.

**Figure 1:**
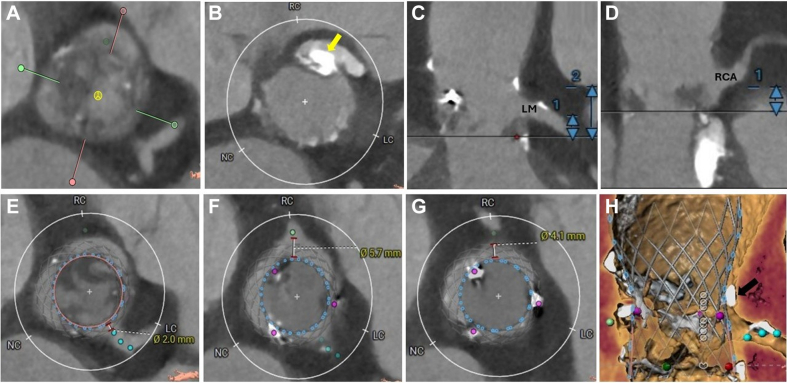
Case 1: Preprocedural Computed Tomography Angiography and Virtual Modeling of the Transcatheter Heart Valve (A to D) Computed tomography angiography showing thickened Mosaic valve leaflets (A), caseous calcification below the right coronary cusp (yellow arrow in B), and relatively low coronary heights from the aortic valve annulus: 6.9 mm for the LM (C) and 7.4 mm for the RCA (D). (E to H) Modeling of a 26-mm Evolut FX+ transcatheter heart valve showed a very short VTC distance of 2 mm (E) for the LM (black arrow in H) and 5.7 mm for the RCA (F), with a VTA distance of 4.1 mm (G). LC = left coronary cusp; LM = left main coronary artery; NC = noncoronary cusp; RC = right coronary cusp; RCA = right coronary artery; VTA = valve to aorta; VTC = valve to coronary.

Pre-TAVR cardiac computed tomography angiography (CTA) showed severely thickened surgical valve leaflets with a caseous calcification below the right coronary cusp. Both coronary heights from the aortic valve annulus were low: 7.4 mm for the right coronary artery (RCA) and 6.9 mm for the left main coronary artery (LM) (Figures 1A to 1D). A 26-mm Evolut FX+ transcatheter heart valve was modeled; the model showed a high risk for LM obstruction, with a virtual valve-to-coronary (VTC) distance of 2 mm and a virtual valve-to–sinotubular junction (VTJ) distance of <2 mm, potentially sequestering the sinuses (Figures 1E to 1H) and prompting the simplified UNICORN leaflet modification technique to prevent iatrogenic occlusion of the LM. The VTC and VTJ distances for the RCA were 5.7 and 4.1 mm, respectively.

**Figure 2:**
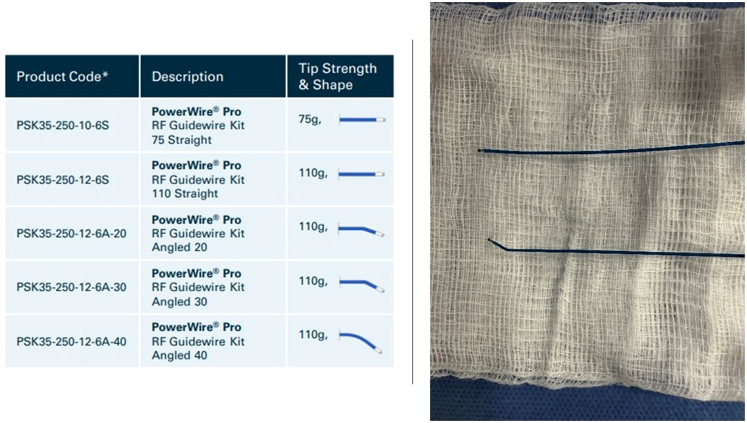
Baylis PowerWire Pro Radiofrequency Wire

**Figure 3:**
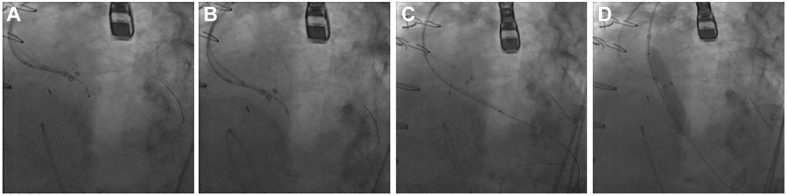
Case 1: Simplified UNICORN of the Left Coronary Leaflet for ViV TAVR (A) Leaflet traversal with a PowerWire Pro 30 RF wire. (B) The NaviCross catheter was advanced into the left ventricle over the PowerWire. (C and D) Leaflet fenestration and laceration with 4-mm and 14-mm balloons, respectively. ViV TAVR = valve-in-valve transcatheter aortic valve replacement.

**Figure 4:**
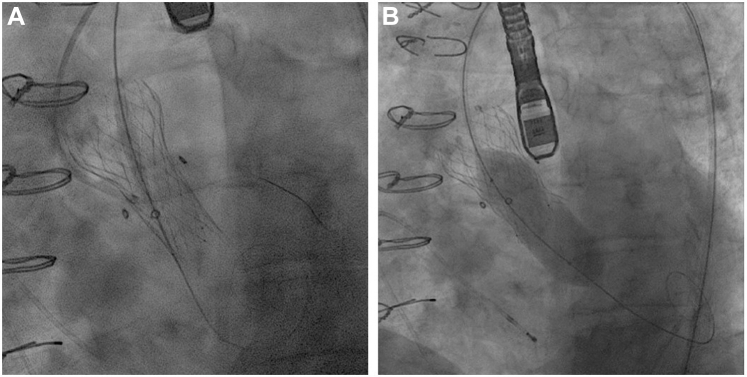
Case 1: Procedural Fluoroscopic Images (A) Successful deployment of 26-mm Evolut FX+ valve, with the valve appearing constrained at inflow. (B) Bicuspid aortic valve postprocedure showing adequate stent frame expansion.

We elected to use the Baylis PowerWire Pro 30 RF wire for leaflet traversal (Figure 2). This wire is similar to the VersaCross J-tip wire. Bilateral groin access was obtained, and an 18-F Gore DrySeal in the left femoral artery and a 12-F DrySeal in the right femoral artery were placed. A 6-F EBU 3.5 guide catheter was used to engage the LM, and a coronary wire was advanced to protect the vessel. Using an AL3 guide catheter and a 135-cm NaviCross catheter (Terumo) in a mother-daughter configuration, the PowerWire Pro 30 was advanced to the left coronary cusp. Under transesophageal echocardiography (TEE) and fluoroscopy guidance (cusp injections), the left leaflet was traversed using the PowerWire Pro 30, using 10 W of energy for 1 second (Figures 3A and 3B). RFP-100 A RF puncture generator (Baylis/Boston Scientific) and energy settings similar to transseptal puncture settings for VersaCross were used. The NaviCross catheter was advanced into the left ventricle, and the PowerWire was exchanged for an XS Safari wire. Leaflet ballooning was performed using 4-mm and 14-mm 0.035-inch wire–compatible peripheral balloons (Armada, Abbott) (Figures 3C and 3D). Following this, a 26-mm Evolut FX+ valve (Medtronic) was deployed within the fenestration created by leaflet ballooning. The constrained portion of the valve was postdilated using a 22-mm True balloon (Bard Medical) (Figures 4A and 4B). Final echocardiogram showed no paravalvular leak and a mean gradient of 10 mm Hg. Aortography showed excellent flow into the LM.

## Case Summary 2

A 76-year-old woman with a history of coronary artery disease, pulmonary hypertension, and diastolic dysfunction presented with heart failure symptoms secondary to severe aortic stenosis. She was deemed unfit for surgery given her history of renal cell cancer with liver metastasis (currently on immunotherapy). TAVR was recommended by the heart team.

**Figure 5:**
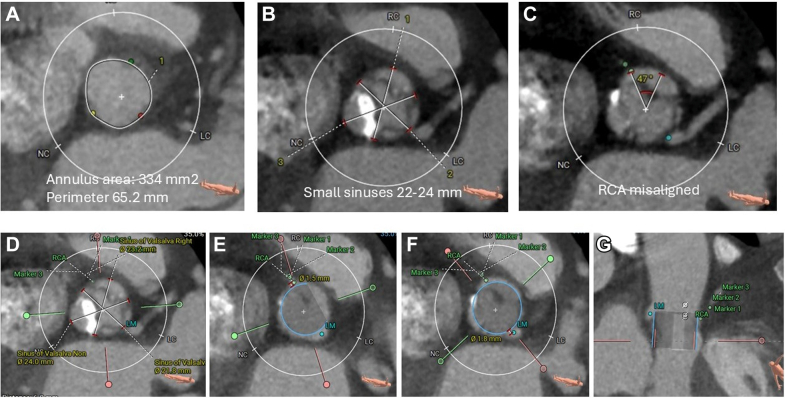
Case 2: Preprocedural Computed Tomography Angiography and Virtual Modeling of the Transcatheter Heart Valve (A to D) Preprocedural computed tomography angiography showing high-risk features: small annulus (A) and sinuses of Valsalva (B), with the RCA arising off axis in the right coronary cusp (C and D). (E to G) VTC distance of <2 mm was found for both the LM and RCA when a 20-mm Sapien S3 virtual valve was modeled. Abbreviations as in Figure 1.

Pre-TAVR CTA demonstrated high-risk features suggesting potential coronary occlusion. Both the right and left coronary leaflets were long, the aortic annulus area was small (334 mm^2^) with a perimeter of 65.2 mm, and the sinuses of Valsalva measured 22 to 24 mm (Figures 5A to 5D). The RCA arose off-center from the right cusp. The VTC and VTJ distances were <2 mm for both the RCA and LM (Figures 5E to 5G). The RCA arose 9.1 mm, and the LM arose 12.7 mm from the aortic valve annulus. A decision was made to perform leaflet modification of the right leaflet using the simplified UNICORN technique and protect the LM with a guide catheter and a coronary wire.

Bilateral groin access was achieved with a 14-F Edwards eSheath in the right common femoral artery and a 12-F Gore DrySeal sheath in the left common femoral artery. Both coronaries were protected by guide catheters and coronary wires. Using an 8-F MP1 guide catheter and NaviCross catheter in a mother-daughter configuration, a PowerWire Pro 30 was advanced to the right coronary cusp. Successful leaflet traversal was performed with 10 W of energy for 1 second. The NaviCross catheter was advanced into the left ventricle, and the PowerWire was exchanged for an XS Safari wire. Leaflet ballooning was performed with 4-mm and 14-mm 0.035-inch wire–compatible peripheral balloons, followed by TAVR with 20-mm Sapien S3 Ultra valve (Edwards Lifesciences). The patient became severely hypotensive immediately postprocedure, and coronary angiography showed severe stenosis of the LM, likely from the long length of the leaflet occluding the ostium, which was not modified before TAVR. This was managed by immediate percutaneous intervention, which restored TIMI flow grade 3 and normalized hemodynamics.See the Visual Summary for intraprocedural images.

The mean post-TAVR gradient was 9 mm Hg without paravalvular leak, and the patient was eventually discharged home in stable condition.

## Procedural Steps for Simplified UNICORN

1. Bilateral groin access with a 14-F or 16-F Edwards eSheath for Sapien S3 valve or an 18-F or 20-F Gore DrySeal sheath for TAVR with Medtronic Evolut FX/FX+ and Abbott Navitor, depending on the valve size.
2. Cerebral embolic protection device use can be considered when the aortic valve leaflets are heavily calcified, although evidence for this in UNICORN and TAVR is lacking.
3. An AL2/3 (LM) or 8-F MP1 (RCA) guide catheter and a 135-cm NaviCross 0.035-inch catheter in a mother-daughter configuration is advanced to the aortic valve.
4. A PowerWire Pro 30 RF wire is positioned at the base and center of the coronary cusp under TEE and fluoroscopy (aortic cusp injections) guidance. Pre-TAVR computed tomography analysis–derived angles can help isolate the right or left coronary cusp.
5. Leaflet traversal is achieved using the PowerWire Pro 30 wire (10 W of energy and 1-second burn), consistent with manufacturer recommendations for the Baylis RF wire system.^5^ Leaflet traversal can be confirmed on TEE. Once the wire traverses the leaflet, the distal position of the wire should be confirmed, and the echocardiogram should be assessed for effusion.
6. Advance the NaviCross into the left ventricle and exchange the PowerWire for an XS Safari wire. Care should be taken not to lose the catheter position during this step.
7. Leaflet fenestration is performed by ballooning the leaflet with 4-mm and 14-mm 0.035-inch–compatible peripheral balloons. A 16-mm balloon may be needed for larger TAVR valves.
8. The TAVR valve should be prepared and ready before leaflet traversal, as there is acute aortic regurgitation through the lacerated leaflets, which can cause acute hemodynamic instability. TAVR is performed as usual over the same XS Safari wire.
9. Post-TAVR ballooning of the valve can be considered if appropriate, especially if the valve stent frame appears constrained on fluoroscopy. This may be particularly true for self-expandable valves.

## Potential Pitfalls and Considerations

The UNICORN procedure presents several technical challenges and limitations that warrant careful consideration. Severely calcified leaflets represent a significant obstacle, potentially precluding balloon passage necessary for leaflet fenestration and laceration. Comprehensive preprocedural CTA and intraprocedural TEE are essential for detailed aortic valve assessment and precise determination of optimal RF wire traversal sites. Calcium-free traversal targets at the leaflet base should be identified, as RF energy cannot effectively penetrate heavily calcified tissue.^6^

An important technical consideration relates to the energy requirements of RF traversal. The lower energy requirements reported here (10 W), compared with the 50 W reported for electrified guidewires in BASILICA, may reflect the optimized electrical properties of purpose-built RF wires, which achieve tissue traversal at lower power thresholds while minimizing char formation and thermal injury. In the original description of UNICORN by Chan et al,^4^ RF pulses were delivered for 1 second using the same VersaCross wire, although the amount of energy used was not specified. Knight et al^7^ compared the VersaCross RF wire and AcQCross needle-dilator system (Medtronic) for transseptal puncture in an ex vivo porcine model and determined that VersaCross RF wire punctures using 10 W of energy for 1 second were associated with higher successful first-attempt punctures, with cleaner cuts, less tissue charring, and no coring.

Leaflet fragment embolization with resultant coronary artery occlusion constitutes a potentially catastrophic complication requiring vigilant monitoring and preparedness for emergent intervention. The technique is optimally suited for cases involving single coronary artery risk; when bilateral coronary compromise is anticipated, hybrid approaches combining UNICORN with complementary leaflet modification strategies should be considered.^8^ Case 2 demonstrates this important limitation. Our approach to performing single-cusp modification was based on a risk assessment that incorporated coronary ostial height in addition to VTC distance. While both coronary arteries had VTC distances of <2 mm, the LM arose at 12.7 mm from the annulus, compared to 9.1 mm for the RCA. Previous multicenter registries have established coronary ostial height thresholds of <12 mm for the LM and <15 mm for the RCA as predictors of increased coronary obstruction risk.^9^ The LM height in case 2 exceeded this threshold, whereas the RCA height did not, which was the basis for our decision. The LM was protected with a guide catheter and was patent on angiographic images taken after UNICORN of the right coronary cusp just before valve deployment. We believe the reason for the coronary obstruction of the LM was possibly the long left leaflet. Given the ample height of the LM from the aortic valve annulus (12.7 mm), we anticipated that protecting the vessel with a guide catheter would be sufficient; however, we lost the guide catheter position while advancing the valve to the aortic valve annulus. Given acute aortic regurgitation from the right leaflet UNICORN, a decision was made to proceed with valve deployment. This highlights the importance of preparing the valve delivery system for rapid implantation before leaflet modification.

Regarding the use of a cerebral embolic protection device, although one was not used in our cases, it can be considered in select cases, especially when leaflets are heavily calcified. Our decision was informed by the PROTECTED TAVR and BHF PROTECT-TAVI trials, which did not demonstrate significant stroke reduction with routine cerebral embolic protection in standard TAVR.^10^ While the BASILICA registry reported a trend toward lower stroke rates with cerebral embolic protection, no definitive conclusions were made owing to low event rates compounded by possible selection bias.^5^ More evidence is needed for a definitive recommendation.

Another limitation of UNICORN is the paucity of robust clinical validation and prospective multicenter registry data compared with the extensively studied BASILICA technique. Large-scale prospective studies are imperative to establish procedural safety, technical success rates, and long-term clinical outcomes.

## Conclusions

This simplified UNICORN technique provides a streamlined approach to leaflet modification during high-risk TAVR that enhances procedural efficiency while preserving technical efficacy and mitigating the risk of hemodynamic instability secondary to acute aortic regurgitation.

**Visual Summary:**
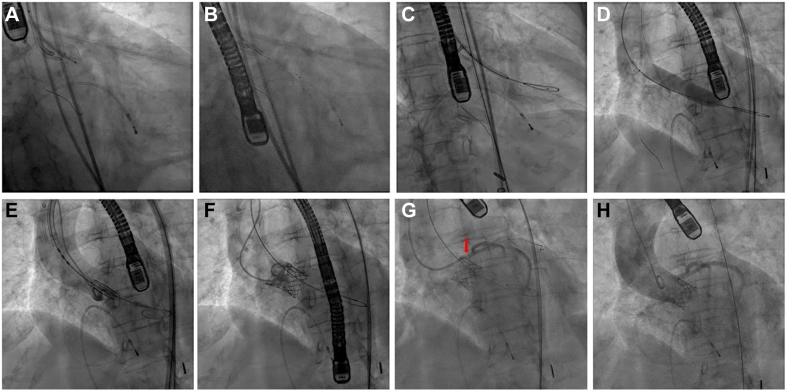
Case 2: Simplified UNICORN of the Right Coronary Leaflet for Native TAVR (A and B) A PowerWire Pro 30 RF wire is advanced to the right coronary cusp through an MP1 guide catheter and NaviCross catheter in a mother-daughter configuration. (C and D) Leaflet ballooning with 4-mm and 14-mm 0.035-inch wire–compatible peripheral balloons. (E and F) Deployment of a 20-mm Sapien S3 valve. (G and H) Ostial left main artery occlusion (red arrow) treated with immediate stenting. TAVR = transcatheter aortic valve replacement.

## Funding Support and Author Disclosures

The authors have reported that they have no relationships relevant to the contents of this paper to disclose.
